# Prediction of Metabolic Syndrome by Non-Alcoholic Fatty Liver Disease in Northern Urban Han Chinese Population: A Prospective Cohort Study

**DOI:** 10.1371/journal.pone.0096651

**Published:** 2014-05-06

**Authors:** Tao Zhang, Yongyuan Zhang, Chengqi Zhang, Fang Tang, Hongkai Li, Qian Zhang, Haiyan Lin, Shuo Wu, Yanxun Liu, Fuzhong Xue

**Affiliations:** 1 Department of Epidemiology and Biostatistics, School of Public Health, Shandong University, Jinan, China; 2 Medical Department,Qilu Hospital of Shandong University, Jinan, China; 3 Health Management Center, Shandong Provincial QianFoShan Hospital, Jinan, China; 4 Center for Health Management, Provincial Hospital affiliated to Shandong University, Jinan, China; Technische Universität Dresden, Medical Faculty, Germany

## Abstract

**Objectives:**

To explore the relationship between non-alcoholic fatty liver disease (NAFLD) and the metabolic syndrome (MetS), and evaluate the value of NAFLD as a marker for predicting the risk of MetS in a large scale prospective cohort from northern urban Han Chinese population.

**Materials and Methods:**

A total of 17,920 MetS-free at baseline cohort members was included in the current study between 2005 and 2011. The baseline characteristics of the cohort were compared by NAFLD status at baseline, MetS status after follow-up. Cox proportional hazards models were used to estimate the unadjusted or adjusted hazard ratios (HRs) for NAFLD at baseline predicting the risk of MetS.

**Results:**

2,183 (12.18%) new cases of MetS occurred between 2005 and 2011. In unadjusted model, HRs (95% CIs) for NAFLD predicting MetS was 3.65 (3.35, 3.97). After adjusting the confounding factors of age, gender, the metabolic factors, smoke and exercise, the HRs (95% CIs) was 1.70 (1.55, 1.87). Gender difference was observed, adjusted HRs (95% CIs) of NAFLD for predicting MetS were 2.06(1.72, 2.46) and 1.55(1.39, 1.72) in female and male population, respectively. Moreover, 163 participants developed MetS among participants without any MetS component at baseline, and its adjusted HRs was still significant, 1.87 (1.12, 3.13).

**Conclusion:**

The present study indicates that NAFLD is an independent risk factor for predicting the risk of MetS in northern urban Han Chinese population, and the people with NAFLD should initiate weight and dietary control to prevent the occurrence of MetS.

## Introduction

There is a growing concern for non-alcoholic fatty liver disease (NAFLD) and metabolic syndrome (MetS). NAFLD is a clinicopathological syndrome that ranges from simple steatosis to steatohepatitis, fibrosis or cirrhosis of the liver [1]. It is associated with dyslipidemia, obesity, and insulin resistance [2], [3], which are the main features of MetS [4]. NAFLD and MetS often are seen in the same individual, whereas insulin resistance probably is a key event that links them together. It is reported that nearly 90% of NAFLD patients has more than one MetS component [5].

The mortality of patients with NAFLD has increased significantly among the general population worldwide [1], [6], and cardiovascular risk competes with liver related risk in dictating the final outcome. It has been reported that MetS is significantly associated with NAFLD as a definite risk factor of cardiovascular disease. A series of cross-sectional studies have reported that NAFLD was linked with MetS and its components [7], [8]. Furthermore, various longitudinal or cohort studies demonstrated that NAFLD was associated with an increased risk of MetS from American in three ethnic groups [9], [10], health survey in western Australia [11], iron workers in Chinese southern population[12], [13], Korean men [2], [14], Japanese [15] and urban south Indians [16].

In the last decade the incidence of NAFLD has been growing in urban Chinese population along with the increasing pandemic of obesity and life-style changes[17], [18], [19], by which the metabolic consequences will significantly burden the health care system of China. Long-term outcomes of NAFLD in Chinese populations remain unclear, and it may be a predictor of MetS, diabetes mellitus and ultimately the cardiovascular disease. Therefore, there are still great needs to evaluate the longitudinal effect of NAFLD on MetS in a large scale prospective cohort, which may be helpful to support the clinical guideline and early intervention strategy for the NAFLD patients before being diagnosed as MetS.

This prospective cohort study was designed to explore the relationship between NAFLD and MetS, and evaluate the value of NAFLD as a marker for predicting the risk of MetS in northern urban Han Chinese population.

## Materials and Methods

### Ethics Statement

The study protocol was approved by ethics committee of School of Public Health, Shandong University. Written informed consent was obtained from all participants.

### Study Population and Cohort Design

Based on the routine health check-up system in Center for Health Management of Shandong Provincial Qianfoshan Hospital and Shandong Provincial Hospital, we set up a large scale longitudinal cohort, and conducted a follow-up from 2005 to 2011 in northern urban Han Chinese population. A total of 28,198 male and female participants who visited the health check-up system for at least two times between 2005 and 2011 were included in this study. Among these participants, 10,278 persons were excluded based on the following exclusion criteria: 6,776 had the alcohol intake regularly; 753 had a positive serologic marker for hepatitis B surface antigen (HBsAg) or hepatitis C virus antibody (HCVAb) at baseline; 4,210 had MetS at baseline; 206 developed MetS before the development of NAFLD during the follow-up period. Because certain participants may have more than one exclusion criteria, the total number of participants who were eligible for this study was 17,920. The average follow-up period was 3.29 (standard deviation, 1.22) years.

### Measurements

The health check-up programs were performed after an overnight fasting of at least 12 hours and all participants underwent a routine anthropometric, clinical, and laboratory tests. The anthropometric measurements involved height, weight, blood pressure. Height and weight were measured while wearing light clothing without shoes. Body mass index (BMI) was calculated as weight (kg) divided by the square of the height (m), and was used as an estimate of obesity. Blood pressure, including systolic blood pressure (SBP) and diastolic blood pressure (DBP), was measured on right arm with participants in a sitting position after a 5-min rest. Blood biochemical analysis includes fasting blood-glucose (FPG), cholesterol, triglycerides (TG), low-density lipoprotein (LDL), high-density lipoprotein (HDL), uric acid, blood urea nitrogen (BUN), serum creatinine (CREA), gamma-glutamyltranspeptidase (GGT), serum albumin (ALB), serum globulins (GLO), white blood count (WBC), hemoglobin (Hb), hematokrit (HCT), mean corpuscular volume(MCV), mean corpuscular hemoglobin (MCH), red blood cell distribution width (RDW), platelet distribution width (PDW), mean platelet volume (MPV), thrombocytocrit (PCT), etc. Moreover, all participants accepted abdominal B-ultrasonograghy examination. Additionally, lifestyle behaviors, including smoking, alcohol intake and physical activity, were surveyed by a questionnaire on health-related behavior.

### Definitions of NAFLD and MetS

According to the revised definition and treatment guidelines for NAFLD by the Chinese Hepatology Association in February 2006 [20], NAFLD was diagnosed by abdominal ultrasonograghy as brightness liver and a diffusely echogenic change in the liver parenchyma, with participants who were diagnosed alcoholic fatty liver disease, infected hepatitis virus (Hepatitis B antigen or hepatitis C antibody positive), and other causes of steatosis excluded.

The diagnostic criteria of metabolic syndrome was from Chinese medical association diabetes branch (CDS) [21], in which MetS was defined as three or more of the following disorders: 1) overweight or obesity (BMI≥25.0 Kg/M^2^); 2) hypertension (SBP≥140 mmHg, DBP ≥90 mmHg or diagnosed before); 3) hyperglycemia (FPG≥6.1 mmol/L or 2 h Postmeal Glucose (PG)≥7.8 mmol/L, or diagnosed before); 4) dyslipidemia (TG≥1.7 mmol/L, or HDL<0.9 mmol/L in male and <1.0 mmol/L in female).

### Statistical Analysis

To account for missing values, multiple imputations were performed. Since imputation method was depended on the patterns of the missing data and the types of the imputed variables, without loss of generality, the Markov chain Monte Carlo (MCMC) method was chosen according to MI Procedure of SAS [22]. Most variables had less than 2% missing observations before imputation except smoking and physical activity having less than 10% missing values.

For the baseline characteristics, continuous variables were summarized by mean (standard deviation) and categorical variables by frequency (percentages %). Their differences, between NAFLD and non-NAFLD groups at baseline, development of MetS during the follow-up and the number of MetS component at baseline, were compared by t-test or F-test for continuous variables, and Chi-square test for categorical variables. The person years were calculated as the sum of follow-up times from the baseline to the occurrence time of MetS or the last health check-up. Cox proportional hazards models (cox-ph) were used to estimate the unadjusted or adjusted hazard ratios (HRs) and 95% confidence intervals (CI) for predicting the risk of MetS by NAFLD at baseline. Totally, 4 cox-ph models were constructed by adjusting different clusters of confounding factors, which might confound the relationship between NAFLD and MetS, including age, gender, the number of MetS component at baseline, obesity, hypertension, hyperglycemia, dyslipidemia, smoking status and regular exercise.

Statistical analysis was performed using SAS version 9.1.3 (SAS Institute, Inc., Cary, NC, USA). A two-sided P<0.05 was considered to be statistically significant.

## Results

During the follow-up between 2005 and 2011, 2,183 (12.18%) new cases of MetS occurred between 2005 and 2011 in the prospective cohort study, and the incidence of MetS (1015 cases, 30.95%) in NAFLD patients was apparently higher than that in non-NAFLD group (1168 cases, 7.98%). The baseline characteristics of the study participants grouped by NAFLD status were shown in Table 1 and Table S1. The baseline prevalence of NAFLD (3,279 cases) was 18.30%. The statistically significant differences between non-NAFLD and NAFLD groups for the listed variables were observed except for MCV, RDW, RDW-SD and PDW.

**Table 1:** Baseline characteristics of participants grouped by NAFLD status.

Characteristic	Non-NAFLD	NAFLD	Total	Statistics*	*P* value
Sample size	14641	3279	17920		
Age (years)	41.50(14.49)	47.02(13.34)	42.51(14.44)	-19.988	<0.0001
Gender				822.737	<0.0001
Male	7075(48.32%)	2491(75.97%)	9566		
Female	7566(51.68%)	788(24.03%)	8354		
Current smoker (%)	2925(19.98%)	1101(33.58%)	4026	284.429	<0.0001
Regular exercise (%)	5257(35.91%)	1257(38.33%)	11406	6.831	0.0090
Number of baseline MetS component				2753.358	<0.0001
none	7463(50.97%)	292(8.91%)	7755		
1	4662(31.84%)	1157(35.29%)	5819		
2	2516(17.18%)	1830(55.81%)	4346		
Obesity (%)	3799(25.95%)	2360(71.97%)	6159	2515.894	<0.0001
Hypertension (%)	2077(14.19%)	795(24.25%)	2872	201.417	<0.0001
Hyperglycemia (%)	685(4.68%)	198(6.04%)	883	10.574	0.0011
Dyslipidemia (%)	3133(21.40%)	1464(44.65%)	4597	759.242	<0.0001

Data are expressed as means (standard deviation) for continuous variables, or frequency (percentages %) for categorical variables.

*Statistics by t-test for continuous variables and Chi-square test for categorical variables.

In contrast to participants without MetS during the follow-up period, those with incident MetS were older, and had a higher prevalence of obesity, hypertension, hyperglycemia and dyslipidemia. The clinical variables (listed in Table S2) demonstrated statistically significant differences between these two groups except for AST, PLT, and PCT. In addition, Table S3 shown the baseline characteristics grouped by the number of MetS components at baseline.

Table 2 presented the HRs (95% CIs) obtained from cox proportional hazards models for the prediction of MetS using NAFLD as the independent variable. In unadjusted model, HRs (95% CIs) for NAFLD predicting MetS was 3.65 (3.35, 3.97). Although the strength of these associations were gradually weakened, HRs still remained statistically significant after adjusting different clusters of potential confounding factors in models 1 to 4. In the cox-ph model 4, the adjusted HRs (95% CIs) was 1.70 (1.55, 1.87).

**Table 2:** Hazard ratios (HRs) and their 95% confidence intervals (CI) from cox model for prediction of MetS using NAFLD as the independent variable.

	Unadjusted	Model 1 1	Model 2 2	Model 3 3	Model 4 4
NAFLD	3.65 (3.35, 3.97)	2.90 (2.66, 3.16)	1.69 (1.55, 1.85)	1.71(1.56, 1.87)	1.70(1.55, 1.87)
Age		1.03 (1.02, 1.03)	1.02 (1.01, 1.02)	1.01(1.01, 1.02)	1.01(1.01, 1.02)
Gender		0.60 (0.55, 0.66)	0.77 (0.70, 0.85)	0.76(0.69, 0.84)	0.8(0.72, 0.89)
No. MetS comp*					
1 vs 0			3.63 (3.04, 4.33)		
2 vs 0			8.81 (7.4, 10.48)		
Obesity				3.21(2.9, 3.56)	3.20(2.89, 3.54)
Hypertension				2.94(2.64, 3.27)	2.94(2.64, 3.28)
Hyperglycemia				3.23(2.8, 3.73)	3.22(2.79, 3.71)
Dyslipidemia				2.09(1.89, 2.31)	2.08(1.88, 2.30)
Smoking status					1.14(1.04, 1.26)
Regular exercise					0.97(0.89, 1.06)

^*^ Number of MetS component at baseline.

1 Adjusted by baseline covariates of age and gender.

2 Adjusted by baseline covariates of age, gender, number of MetS component.

3 Adjusted by baseline covariates of age, gender, obesity, hypertension, hyperglycemia and dyslipidemia.

4 Adjusted by baseline covariates of age, gender, obesity, hypertension, hyperglycemia, dyslipidemia, smoking status and regular exercise.

Note that the gender difference was observed in models 1 to 4, the further subgroup analyses for female and male population were conducted (shown in Table S4 and Table S5). In unadjusted model, HRs (95% CIs) for NAFLD predicting MetS were 6.52(5.54, 7.68) and 2.44(2.21, 2.69) in female and male population, respectively. After adjusting the confounding factors, HRs (95% CIs) decreased to 2.06(1.72, 2.46) and 1.55(1.39, 1.72) in female and male population, respectively.

In addition, among 7,755 participants free of any MetS component at baseline, 163(7.47%) developed MetS during the follow-up period (shown in Table S3). Table 3 presented that HRs (95% CIs) for NAFLD predicting MetS in this sub-population were 3.46 (2.12, 5.64) and 1.87 (1.12, 3.13) in the unadjusted and adjusted model.

**Table 3:** Hazard ratios (HRs) and their 95% confidence intervals (CI) from cox-ph model (NAFLD predicting MetS) in the participants free of any MetS component at baseline.

	Unadjusted	Model 1 1	Model 2 2	Model 3 3
NAFLD	3.46 (2.12, 5.64)	2.49 (1.51, 4.10)	1.83 (1.10, 3.06)	1.87 (1.12, 3.13)
Age		1.01 (1.00, 1.03)	1.01 (0.99, 1.02)	1.00 (0.99, 1.02)
Gender		0.48 (0.35, 0.66)	0.64 (0.45, 0.92)	0.70 (0.48, 1.03)
BMI			1.17 (1.06, 1.29)	1.16 (1.06, 1.28)
Systolic BP			1.02 (1.00, 1.03)	1.02 (1.00, 1.03)
Fasting serum glucose			1.49 (1.09, 2.04)	1.52 (1.11, 2.07)
GGT			1.00 (1.00, 1.01)	1.00 (1.00, 1.01)
Total cholesterol			1.07 (0.72, 1.59)	1.08 (0.73, 1.61)
BUN			0.99 (0.86, 1.14)	0.98 (0.85, 1.13)
Smoking status				1.10 (0.74, 1.62)
Regular exercise				0.67 (0.48, 0.92)

1 Adjusted by baseline covariates of age and gender.

2 Adjusted by baseline covariates of age, gender, systolic BP, fasting serum glucose, GGT, total cholesterol and BUN.

3 Adjusted by baseline covariates of age, gender, systolic BP, fasting serum glucose, GGT, total cholesterol, BUN, smoking status and regular exercise at baseline.

## Discussion

In this prospective cohort study, 2,183 (12.18%) new cases of MetS occurred during the follow-up period between 2005 and 2011, and the incidence of MetS in NAFLD patients was apparently higher than that in non-NAFLD group. The Cox proportional hazards models demonstrated that NAFLD was shown to be an independent risk factor for MetS after adjusting for the potential confounding factors. To the best of our knowledge, this study is the first large scale prospective cohort study showing the availability of NAFLD as a predictor for the risk of MetS in the urban Han Chinese population. Similar results were found in other national and regional population, such as American in three ethnic groups [9], [10], health survey from western Australia [11], iron workers from Chinese southern population [12], [13], Korean men [2], [14], Japanese [15] and urban south Indians [16].

The gender difference for the association between NAFLD and MetS was observed in cox proportional hazards models, which is an interesting point of this study corresponding to our study aim. The prevalence of NAFLD in male (2491/9566, 26.04%) is obviously higher than that in female population (788/8354, 9.43%), and the possibility that female hormones protect against NAFLD has been postulated [23]. However, adjusted HRs (95% CIs) of NAFLD for predicting MetS were 2.06(1.72, 2.46) and 1.55(1.39, 1.72) in female and male population, respectively. This indicates that the female population with NAFLD has a higher risk for MetS in future than that in males. Therefore, more attention should be paid to the female population with NAFLD in the clinical guideline.

In addition, the potential confounding factors should be included as the covariates in Cox proportional hazards models, which is very important to construct an effective model for evaluating the association between NAFLD and MetS. As expected, the metabolic factors and the number of MetS components at baseline were significant risk factors of MetS [4], [24]. After adjusting for the multiple covariates listed in Table 2, HRs for MetS was still higher in NAFLD group than normal group. Moreover, 163 participants developed MetS during the follow-up period among participants without any MetS component at baseline, and the HRs specific to this pure population was still significant. Furthermore, the participants who developed MetS before the development of NAFLD were excluded in our prospective cohort study. The above evidences might indicate that NAFLD was a potential independent causal factor for predicting the risk of MetS in northern urban Han Chinese population.

As for the mechanisms of these results, the association between NAFLD and MetS could be mainly explained by insulin resistance (IR), which was the main mechanism linking NAFLD and MetS from pathophysiological perspective [25]. Many researches demonstrated that IR was a pathogenic factor of the metabolic syndrome and also played a major role in the development and progression of NAFLD [1], [26]. In NAFLD population, excess fat accumulation in the hepatic parenchyma or overabundance of fatty acids was a major contributor to the development of IR, and further caused MetS [4], [27], [28]. And NAFLD commonly manifested to be nonalcoholic steatohepatitis (NASH), which was the result of “two hits” and might result in abnormal lipid metabolism, increased oxidative stress, and accelerated progression of MetS [5], [29]. Moreover, NAFLD involved hepatic lipid peroxidation, which activated inflammatory cytokines, such as interleukin 6, tumor necrosis factor (TNF) and C-reactive protein (CRP) [30], [31], [32], [33], and led to IR, then MetS. In addition, Hypoadiponentinemia was proposed to be a feature of NAFLD [34] and associated with obesity, hyperlipidemia and hyperglycemia, which were components of MetS and characterized by IR [35].

Although the debate whether NAFLD should be included as one of the components of the MetS is still in existence [9], [36], the conclusion can be drawn that NAFLD was an important independent risk factor for prediction of MetS. The participants in our study were from the medical health check-up program, whose purpose is to promote the health and enhance early detection of existing diseases. The result of our study might be helpful to support the clinical guideline and early intervention strategy for the NAFLD patients before being diagnosed as MetS [37], [38]. Therefore, as for the NAFLD diagnosed in the health check-up, NAFLD could help initiate weight and dietary control at the “earliest possible time” in the progression of disease, so as to prevent the development of MetS, diabetes mellitus, and even the cardiovascular diseases.

Nevertheless, there seems to be some limitations in our study. First, the presence of NAFLD was assessed by abdominal ultrasonograghy by experienced radiologists instead of pathologic finding, and we have no information on the intra- or inter-observer reliability of ultrasonographic examinations. Second, owing to the absence of waist circumference measurement in the health check-up program, the diagnostic criteria of MetS was based on the Chinese medical association diabetes branch, rather than the international Standard criteria. Third, the present study was based on the routine health check-up system in northern urban Han Chinese population of Shandong province, further investigation need to be done to confirm the value of NAFLD as a marker for predicting the risk of MetS in a larger area of general population.

## Supporting Information

Table S1 Baseline clinical characteristics of participants grouped by NAFLD status.
(DOC)

Table S2 Characteristics comparison between participants with and without incident MetS during the follow-up period.
(DOC)

Table S3 Baseline characteristics of the study participants according to the number of baseline MetS-free components.
(DOC)

Table S4 Hazard ratios (HRs) and their 95% confidence intervals (CI) from cox model (NAFLD predicting Mets) in the female population.
(DOC)

Table S5 Hazard ratios (HRs) and their 95% confidence intervals (CI) from cox model (NAFLD predicting Mets) in the male population.
(DOC)
