# Supplementary material for: Prediction of Metabolic Syndrome by Non-Alcoholic Fatty Liver Disease in Northern Urban Han Chinese Population: A Prospective Cohort Study
Source: PLoS One. 2014 May 6;9(5):e96651. doi: 10.1371/journal.pone.0096651 (PMC4011868; doi:10.1371/journal.pone.0096651)
Supplement: Table S1 — Baseline clinical characteristics of participants grouped by NAFLD status. (DOC) [file pone.0096651.s001.doc]

**Table S1** Baseline clinical characteristics of participants grouped by NAFLD status.

| **Characteristics** | **Non-NAFLD** | **NAFLD** | **Total** | **Statistics*** | ***P* value** |
| --- | --- | --- | --- | --- | --- |
| Sample size | 14641 | 3279 | 17920 |  |  |
| BMI(kg/m^2^) | 23.21(2.96) | 26.70(2.72) | 23.85(3.21) | -61.969 | <0.0001 |
| Systolic BP (mmHg) | 119.00(17.42) | 128.27(16.44) | 120.70(17.61) | -27.805 | <0.0001 |
| Diastolic BP (mmHg) | 70.42(10.27) | 76.48(10.05) | 71.53(10.50) | -30.608 | <0.0001 |
| Fasting serum glucose (mg/dL) | 4.92(0.82) | 5.18(0.84) | 4.97(0.83) | -15.957 | <0.0001 |
| Total cholesterol (mg/dL) | 4.86(0.94) | 5.21(0.94) | 4.92(0.95) | -19.091 | <0.0001 |
| Triglyceride (mg/dL) | 1.10(0.86) | 1.79(1.26) | 1.22(0.98) | -37.989 | <0.0001 |
| HDL-cholesterol (mg/dL) | 1.37(0.33) | 1.24(0.30) | 1.35(0.32) | 21.318 | <0.0001 |
| LDL-cholesterol (mg/dL) | 2.74(0.73) | 3.08(0.72) | 2.80(0.74) | -23.662 | <0.0001 |
| ALT(U/L) | 16.22(12.25) | 26.14(28.48) | 18.05(16.95) | -29.261 | <0.0001 |
| AST(U/L) | 21.07(8.03) | 22.96(7.13) | 21.66(7.80) | -2.212 | 0.0275 |
| GGT(U/L) | 18.84(16.41) | 30.64(25.38) | 21.00(18.94) | -33.251 | <0.0001 |
| BUN(mg/L) | 4.85(1.24) | 5.26(1.34) | 4.93(1.27) | -16.471 | <0.0001 |
| CREA(mg/L) | 78.55(13.93) | 84.30(16.32) | 79.60(14.57) | -20.684 | <0.0001 |
| RBC(10^9^g/L) | 4.80(0.47) | 5.06(0.43) | 4.85(0.47) | -24.215 | <0.0001 |
| HCT (%) | 43.05(3.95) | 45.35(3.43) | 43.47(3.96) | -30.806 | <0.0001 |
| MCV(fL) | 89.88(4.69) | 89.72(4.04) | 89.85(4.58) | 1.868 | 0.0618 |
| MCH(pg) | 29.96(1.97) | 30.16(1.61) | 29.99(1.91) | -5.353 | <0.0001 |
| MCHC(g/L) | 333.19(11.46) | 336.14(10.51) | 333.73(11.35) | -13.532 | <0.0001 |
| RDW(%) | 12.81(1.01) | 12.79(0.73) | 12.80(0.97) | 0.933 | 0.3508 |
| RDW-SD(fL) | 41.34(2.59) | 41.31(2.47) | 41.34(2.57) | 0.665 | 0.5058 |
| WBC(10^9^g/L) | 6.30(1.51) | 6.97(1.58) | 6.42(1.55) | -22.149 | <0.0001 |
| PLT(%) | 236.90(53.94) | 241.98(55.52) | 237.84(54.27) | -4.450 | <0.0001 |
| PDW(%) | 12.31(1.94) | 12.29(1.71) | 12.31(1.90) | 0.554 | 0.5794 |
| MPV(fL) | 10.42(0.82) | 10.36(0.80) | 10.41(0.82) | 3.941 | 0.0001 |
| PCT(%) | 0.25(0.07) | 0.25(0.07) | 0.25(0.07) | -2.136 | 0.0327 |

Data are means (standard deviation) for continuous variables, or percentages for categorical variables.

*Statistics by t-test for continuous variables and Chi square test for categorical variables.
