# Supplementary material for: Prediction of Metabolic Syndrome by Non-Alcoholic Fatty Liver Disease in Northern Urban Han Chinese Population: A Prospective Cohort Study
Source: PLoS One. 2014 May 6;9(5):e96651. doi: 10.1371/journal.pone.0096651 (PMC4011868; doi:10.1371/journal.pone.0096651)
Supplement: Table S2 — Characteristics comparison between participants with and without incident MetS during the follow-up period. (DOC) [file pone.0096651.s002.doc]

**Table S2** Characteristics comparison between participants with and without incident MetS during the follow-up period.

| **Characteristics** | **MetS free** | **Mets** | **Total** | **Statistics*** | ***P* value** |
| --- | --- | --- | --- | --- | --- |
| Sample size | 15737 | 2183 | 17920 |  |  |
| NAFLD at baseline (%) | 2264(14.39%) | 1015(46.50%) | 3279 | 1322.086 | <0.0001 |
| Age at baseline | 41.27(13.91) | 51.49(15.04) | 42.51(14.44) | -31.850 | <0.0001 |
| Gender |  |  |  | 369.186 | <0.0001 |
| Male | 7981(50.71%) | 1585(72.61%) | 9566 |  |  |
| Female | 7756(49.29%) | 598(27.39%) | 8354 |  |  |
| BMI(kg/m^2^) | 23.47(3.06) | 26.61(2.89) | 23.85(3.21) | -47.298 | <0.0001 |
| Obesity (%) | 4620(29.36%) | 1539(70.50%) | 6159 | 1438.549 | <0.0001 |
| Hypertension (%) | 2002(12.72%) | 870(39.85%) | 2872 | 1048.595 | <0.0001 |
| Hyperglycemia (%) | 636(4.04%) | 247(11.31%) | 883 | 216.481 | <0.0001 |
| Dyslipidemia (%) | 3806(24.19%) | 791(36.23%) | 4597 | 145.940 | <0.0001 |
| No. Mets comp |  |  |  | 2490.934 | <0.0001 |
| none | 7592(48.24%) | 163(7.47%) | 7755 |  |  |
| 1 | 5226(33.21%) | 593(27.16%) | 5819 |  |  |
| 2 | 2919(18.55%) | 1427(65.37%) | 4346 |  |  |
| Systolic BP (mmHg) | 118.78(16.54) | 134.49(18.91) | 120.70(17.61) | -40.824 | <0.0001 |
| Diastolic BP (mmHg) | 70.64(10.10) | 77.94(11.05) | 71.53(10.50) | -31.264 | <0.0001 |
| Fasting serum glucose (mg/dL) | 4.91(0.76) | 5.42(1.14) | 4.97(0.83) | -27.540 | <0.0001 |
| ALT(U/L) | 17.44(17.16) | 22.27(14.75) | 18.05(16.95) | -11.989 | <0.0001 |
| AST(U/L) | 21.50(7.85) | 22.48(7.56) | 21.66(7.80) | -0.907 | 0.3647 |
| GGT(U/L) | 19.95(18.25) | 28.52(21.85) | 21.00(18.94) | -20.019 | <0.0001 |
| BUN(mg/L) | 4.86(1.22) | 5.38(1.52) | 4.93(1.27) | -18.094 | <0.0001 |
| CREA(mg/L) | 78.94(13.92) | 84.37(17.92) | 79.60(14.57) | -16.435 | <0.0001 |
| Total cholesterol (mg/dL) | 4.88(0.92) | 5.22(1.09) | 4.92(0.95) | -15.829 | <0.0001 |
| Triglyceride (mg/dL) | 1.17(0.95) | 1.63(1.08) | 1.22(0.98) | -20.817 | <0.0001 |
| HDL-cholesterol (mg/dL) | 1.36(0.33) | 1.27(0.30) | 1.35(0.32) | 12.847 | <0.0001 |
| LDL-cholesterol (mg/dL) | 2.77(0.72) | 3.08(0.80) | 2.80(0.74) | -18.744 | <0.0001 |
| RBC(10^9^g/L) | 4.83(0.47) | 5.00(0.44) | 4.85(0.47) | -14.324 | <0.0001 |
| HCT (%) | 43.26(3.97) | 44.97(3.59) | 43.47(3.96) | -19.143 | <0.0001 |
| MCV(fL) | 89.83(4.60) | 90.03(4.43) | 89.85(4.58) | -2.023 | 0.0432 |
| MCH(pg) | 29.97(1.93) | 30.18(1.79) | 29.99(1.91) | -4.897 | <0.0001 |
| MCHC(g/L) | 333.52(11.43) | 335.21(10.63) | 333.73(11.35) | -6.534 | <0.0001 |
| RDW(%) | 12.79(0.98) | 12.87(0.88) | 12.80(0.97) | -3.635 | 0.0003 |
| RDW-SD(fL) | 41.29(2.56) | 41.65(2.62) | 41.34(2.57) | -6.206 | <0.0001 |
| WBC(10^9^g/L) | 6.36(1.53) | 6.89(1.61) | 6.42(1.55) | -14.626 | <0.0001 |
| PLT(%) | 237.77(53.94) | 238.27(56.47) | 237.84(54.27) | -0.364 | 0.7158 |
| PDW(%) | 12.32(1.93) | 12.21(1.68) | 12.31(1.90) | 2.428 | 0.0152 |
| MPV(fL) | 10.42(0.82) | 10.33(0.80) | 10.41(0.82) | 5.078 | <0.0001 |
| PCT(%) | 0.25(0.07) | 0.25(0.07) | 0.25(0.07) | 0.399 | 0.6899 |
| Current smoker (%) | 3350(21.29%) | 676(30.97%) | 4026 | 103.107 | <0.0001 |
| Regular exercise (%) | 5470(34.76%) | 1044(47.82%) | 6514 | 141.439 | <0.0001 |

Data are means (standard deviation) for continuous variables, or percentages for categorical variables.

*Statistics by t-test for continuous variables and Chi square test for categorical variables.
