# Supplementary material for: Prediction of Metabolic Syndrome by Non-Alcoholic Fatty Liver Disease in Northern Urban Han Chinese Population: A Prospective Cohort Study
Source: PLoS One. 2014 May 6;9(5):e96651. doi: 10.1371/journal.pone.0096651 (PMC4011868; doi:10.1371/journal.pone.0096651)
Supplement: Table S3 — Baseline characteristics of the study participants according to the number of baseline MetS-free components. (DOC) [file pone.0096651.s003.doc]

**Table S3** Baseline characteristics of the study participants according to the number of baseline MetS-free components

| **Characteristics** | **Number of MetS component at baseline** | | | **Total** | ***P* value** |
| --- | --- | --- | --- | --- | --- |
|  | **1** | **2** |  |  |  |
| Sample size | 7755 | 5819 | 4346 | 17920 |  |
| NAFLD at baseline (%) | 292(3.77%) | 1157(19.88%) | 1830(42.11%) | 3279 | <0.0001 |
| Age at baseline | 37.98(11.99) | 43.92(14.71) | 48.72(15.37) | 42.51(14.44) | <0.0001 |
| Gender |  |  |  |  | <0.0001 |
| Male | 2886(37.21%) | 3474(59.70%) | 3206(73.77%) | 9566 |  |
| Female | 4869(62.79%) | 2345(40.30%) | 1140(26.23%) | 8354 |  |
| BMI(kg/m^2^) | 21.70(2.04) | 24.53(2.90) | 26.78(2.53) | 23.85(3.21) | <0.0001 |
| Obesity (%) | 0(0.00%) | 2567(44.11%) | 3592(82.65%) | 6159 | <0.0001 |
| Hypertension (%) | 0(0.00%) | 988(16.98%) | 1884(43.35%) | 2872 | <0.0001 |
| Hyperglycemia (%) | 0(0.00%) | 315(5.41%) | 568(13.07%) | 883 | <0.0001 |
| Dyslipidemia (%) | 0(0.00%) | 1949(33.49%) | 2648(60.93%) | 4597 | <0.0001 |
| Systolic BP (mmHg) | 111.84(11.78) | 122.85(16.46) | 133.63(18.86) | 120.70(17.61) | <0.0001 |
| Diastolic BP (mmHg) | 67.17(8.55) | 72.57(9.76) | 77.92(10.96) | 71.53(10.50) | <0.0001 |
| Fasting serum glucose (mg/dL) | 4.74(0.53) | 5.02(0.86) | 5.30(1.07) | 4.97(0.83) | <0.0001 |
| ALT(U/L) | 14.51(11.85) | 19.14(21.90) | 22.86(15.75) | 18.05(16.95) | <0.0001 |
| AST(U/L) | 20.13(5.09) | 21.35(6.24) | 23.25(10.40) | 21.66(7.80) | <0.0001 |
| GGT(U/L) | 16.17(12.51) | 21.98(21.05) | 28.29(22.59) | 21.00(18.94) | <0.0001 |
| BUN(mg/L) | 4.68(1.16) | 5.01(1.26) | 5.25(1.38) | 4.93(1.27) | <0.0001 |
| CREA(mg/L) | 75.79(12.54) | 80.83(14.53) | 84.75(16.06) | 79.60(14.57) | <0.0001 |
| Total cholesterol (mg/dL) | 4.73(0.87) | 4.98(0.93) | 5.19(1.04) | 4.92(0.95) | <0.0001 |
| Triglyceride (mg/dL) | 0.78(0.50) | 1.34(0.90) | 1.87(1.29) | 1.22(0.98) | <0.0001 |
| HDL-cholesterol (mg/dL) | 1.46(0.29) | 1.31(0.32) | 1.22(0.33) | 1.35(0.32) | <0.0001 |
| LDL-cholesterol (mg/dL) | 2.62(0.69) | 2.86(0.71) | 3.06(0.76) | 2.80(0.74) | <0.0001 |
| RBC(10^9^g/L) | 4.72(0.45) | 4.89(0.46) | 5.03(0.45) | 4.85(0.47) | <0.0001 |
| HCT (%) | 42.29(3.86) | 43.87(3.85) | 45.02(3.64) | 43.47(3.96) | <0.0001 |
| MCV(fL) | 89.89(4.78) | 89.85(4.41) | 89.77(4.43) | 89.85(4.58) | <0.0001 |
| MCH(pg) | 29.89(2.04) | 30.03(1.82) | 30.13(1.79) | 29.99(1.91) | <0.0001 |
| MCHC(g/L) | 332.33(11.41) | 334.15(11.20) | 335.64(11.11) | 333.73(11.35) | <0.0001 |
| RDW(%) | 12.79(1.05) | 12.79(0.92) | 12.84(0.88) | 12.80(0.97) | <0.0001 |
| RDW-SD(fL) | 41.27(2.52) | 41.31(2.57) | 41.49(2.64) | 41.34(2.57) | <0.0001 |
| WBC(10^9^g/L) | 6.11(1.46) | 6.48(1.53) | 6.90(1.59) | 6.42(1.55) | <0.0001 |
| PLT(%) | 237.68(53.41) | 237.57(53.96) | 238.45(56.13) | 237.84(54.27) | 0.5337 |
| PDW(%) | 12.32(1.71) | 12.29(1.75) | 12.30(2.38) | 12.31(1.90) | 0.4368 |
| MPV(fL) | 10.46(0.81) | 10.39(0.82) | 10.35(0.82) | 10.41(0.82) | <0.0001 |
| PCT(%) | 0.25(0.07) | 0.25(0.07) | 0.25(0.07) | 0.25(0.07) | 0.0373 |
| Current smoker (%) | 1204(15.53%) | 1460(25.09%) | 1362(31.34%) | 4026.00 | <0.0001 |
| Regular exercise (%) | 2393(30.86%) | 2285(39.27%) | 1836(42.25%) | 6514.00 | <0.0001 |
| Development of MetS (%) | 163(7.47%) | 593(27.16%) | 1427(65.37%) | 2490.93 | <0.0001 |
| Development of MetS  by NAFLD status (%) |  |  |  |  |  |
| Non-NAFLD | 145(1.94%) | 368(7.89%) | 655(26.03%) | 1168(7.98%) | <0.0001 |
| NAFLD | 18(6.16%) | 225(19.45%) | 772(42.19%) | 1.15(30.95%) | <0.0001 |

Data are means (standard deviation) for continuous variables, or percentages for categorical variables.

*Statistics by F-test for continuous variables and Chi square test for categorical variables.
