# Supplementary material for: Prediction of Metabolic Syndrome by Non-Alcoholic Fatty Liver Disease in Northern Urban Han Chinese Population: A Prospective Cohort Study
Source: PLoS One. 2014 May 6;9(5):e96651. doi: 10.1371/journal.pone.0096651 (PMC4011868; doi:10.1371/journal.pone.0096651)
Supplement: Table S4 — Hazard ratios (HRs) and their 95% confidence intervals (CI) from cox model (NAFLD predicting Mets) in the female population. (DOC) [file pone.0096651.s004.doc]

**Table S4** Hazard ratios (HRs) and their 95% confidence intervals (CI) from cox model (NAFLD predicting Mets) in the female population.

|  | Hazard ratios (95% confidence interval) | | | | |
| --- | --- | --- | --- | --- | --- |
|  | Unadjusted | Model 1 ^1^ | Model 2 ^2^ | Model 3 ^3^ | Model 4 ^4^ |
| NAFLD | 6.52(5.54,7.68) | 3.90(3.29,4.62) | 2.05(1.72,2.45) | 2.05(1.71,2.46) | 2.06(1.72,2.46) |
| Age |  | 1.05(1.05,1.06) | 1.03(1.03,1.04) | 1.03(1.02,1.04) | 1.03(1.02,1.04) |
| No. Mets comp^*^ |  |  |  |  |  |
| 1 vs 0 |  |  | 3.48(2.61,4.63) |  |  |
| 2 vs 0 |  |  | 9.25(6.93,12.34) |  |  |
| Obesity |  |  |  | 3.4(2.83,4.08) | 3.4(2.83,4.08) |
| Hypertension |  |  |  | 3.06(2.51,3.74) | 3.06(2.51,3.74) |
| Hyperglycemia |  |  |  | 3.5(2.7,4.54) | 3.52(2.72,4.57) |
| Dyslipidemia |  |  |  | 2.15(1.77,2.61) | 2.13(1.76,2.59) |
| Smoking status |  |  |  |  | 1.32(0.9,1.93) |
| Regular exercise |  |  |  |  | 0.99(0.84,1.18) |

^*^ Number of MetS component at baseline.

^1^ Adjusted by baseline covariates of age and gender.

^2^ Adjusted by baseline covariates of age, gender, number of MetS component.

^3^ Adjusted by baseline covariates of age, gender, obesity, hypertension, hyperglycemia and dyslipidemia.

^4^ Adjusted by baseline covariates of age, gender, obesity, hypertension, hyperglycemia, dyslipidemia, smoking status and regular exercise.
