# Supplementary material for: Prediction of Metabolic Syndrome by Non-Alcoholic Fatty Liver Disease in Northern Urban Han Chinese Population: A Prospective Cohort Study
Source: PLoS One. 2014 May 6;9(5):e96651. doi: 10.1371/journal.pone.0096651 (PMC4011868; doi:10.1371/journal.pone.0096651)
Supplement: Table S5 — Hazard ratios (HRs) and their 95% confidence intervals (CI) from cox model (NAFLD predicting Mets) in the male population. (DOC) [file pone.0096651.s005.doc]

**Table S5** Hazard ratios (HRs) and their 95% confidence intervals (CI) from cox model (NAFLD predicting Mets) in the male population.

|  | Hazard ratios (95% confidence interval) | | | | |
| --- | --- | --- | --- | --- | --- |
|  | Unadjusted | Model 1 ^1^ | Model 2 ^2^ | Model 3 ^3^ | Model 4 ^4^ |
| NAFLD | 2.44(2.21,2.69) | 2.4(2.18,2.65) | 1.54(1.39,1.70) | 1.55(1.4,1.72) | 1.55(1.39,1.72) |
| Age |  | 1.02(1.02,1.02) | 1.01(1.01,1.01) | 1.01(1.00,1.01) | 1.01(1.00,1.01) |
| No. Mets comp^*^ |  |  |  |  |  |
| 1 vs 0 |  |  | 3.18(2.54,3.98) |  |  |
| 2 vs 0 |  |  | 7.23(5.81,8.99) |  |  |
| Obesity |  |  |  | 2.94(2.60,3.32) | 2.92(2.59,3.30) |
| Hypertension |  |  |  | 2.69(2.37,3.06) | 2.70(2.38,3.07) |
| Hyperglycemia |  |  |  | 2.93(2.47,3.47) | 2.90(2.45,3.44) |
| Dyslipidemia |  |  |  | 1.91(1.69,2.15) | 1.90(1.69,2.14) |
| Smoking status |  |  |  |  | 1.12(1.01,1.24) |
| Regular exercise |  |  |  |  | 0.97(0.87,1.07) |

^*^ Number of MetS component at baseline.

^1^ Adjusted by baseline covariates of age and gender.

^2^ Adjusted by baseline covariates of age, gender, number of MetS component.

^3^ Adjusted by baseline covariates of age, gender, obesity, hypertension, hyperglycemia and dyslipidemia.

^4^ Adjusted by baseline covariates of age, gender, obesity, hypertension, hyperglycemia, dyslipidemia, smoking status and regular exercise.
